# teen Mental Health First Aid: 12-month outcomes from a cluster crossover randomized controlled trial evaluation of a universal program to help adolescents better support peers with a mental health problem

**DOI:** 10.1186/s12889-022-13554-6

**Published:** 2022-06-10

**Authors:** Laura M. Hart, Amy J. Morgan, Alyssia Rossetto, Claire M. Kelly, Karen Gregg, Maxine Gross, Catherine Johnson, Anthony F. Jorm

**Affiliations:** 1 Centre for Mental Health, Melbourne School of Population and Global Health , University of Melbourne, Melbourne, Australia; 2 School of Psychology and Public Health, La Trobe University, Melbourne, Australia; 3 Mental Health First Aid Australia, Melbourne, Australia

**Keywords:** Mental Health First Aid, Mental health literacy, Stigma, Adolescents, Help-seeking

## Abstract

**Background:**

teen Mental Health First Aid (tMHFA) is a universal mental health literacy, stigma reduction, help-seeking, and suicide prevention program designed for adolescents in Years 10–12 of secondary school (16–18 years). tMHFA is delivered by trained instructors, in a regular classroom setting, to increase the knowledge, attitudes and behaviours that adolescents’ require to better support peers with mental health problems or mental health crises.

**Methods:**

To explore the efficacy of tMHFA, a cluster crossover randomised controlled trial was conducted with Year 10 students in four schools in Victoria, Australia, using physical first aid training as the control intervention. Of the 1942 eligible students, 1,624 completed baseline and 894 completed follow-up surveys. Online surveys, administered one week before training and again 12-months later, included vignettes depicting peers John (depression and suicide risk) and Jeanie (social anxiety/phobia), measures of mental health first aid (quality of first aid intentions, confidence, first aid behaviours provided, and first aid behaviours received), mental health literacy (beliefs about adult help, help-seeking intentions), and stigma (social distance, weak-not-sick, dangerous/unpredictable, and would not tell anyone).

**Results:**

The primary outcome—quality of first aid intentions towards the John vignette—showed statistically significant group x time interactions, with tMHFA students reporting more helpful and less unhelpful first aid intentions, than PFA students did over time. Confidence in providing first aid also showed significant interactions. First aid behaviours—both those provided to a peer with a mental health problem and those received from a peer—showed null results. Ratings of both beliefs about adult help and help-seeking intentions were found to be significantly improved among tMHFA students at follow-up. A group x time interaction was found on one stigma scale (would not tell anyone).

**Conclusions:**

This trial showed that, one year after training, tMHFA improves first aid intentions towards peers with depression and suicide risk, confidence in helping peers with mental health problems, willingness to tell someone and seek help from an adult or health professional if experiencing a mental health problem.

**Trial registration:**

This research was registered with Australia New Zealand Clinical Trials Registry: ACTRN12614000061639.

**Supplementary Information:**

The online version contains supplementary material available at 10.1186/s12889-022-13554-6.

## Background

Mental health problems (MHPs), such as anxiety, depression and their sub-clinical symptoms, are a leading cause of disability among young people [1]. MHPs not only cause immediate distress for adolescents, they also often disrupt important social, physical and psychological milestones necessary for healthy transition into adulthood [2]. Even though approximately half of all mental illnesses experienced across the lifespan present by mid-adolescence [3, 4], few adolescents receive treatment [5]. For young people who do access mental health care, there is often a delay between presentation of symptoms and seeking professional help, which complicates treatment outcomes and prognosis [6]. Adolescents are, however, more likely to seek initial help from informal sources such as friends or family [7, 8]. This presents an opportunity for social contacts to facilitate a young person’s access to appropriate mental health care. Yet, for this to occur it is important that the people who surround teenagers have the knowledge and skills to recognise and appropriately respond to MHPs and crises [8].

The provision of mental health literacy programs in secondary schools has proliferated over the last decade, but evaluation research typically involves uncontrolled trials with short or no follow-up measurement [9, 10]. Without high-quality controlled trials with longer-term follow-up, it is impossible to tell whether short-term improvements to knowledge or attitudes are sustained and thereby likely to improve early recognition and appropriate help-seeking among young people and across the course of adolescence [11]. The current paper reports on outcomes at 12-month follow up of a cluster cross over (CRXO) randomised controlled trial comparing a Mental Health First Aid (MHFA) training program to a matched emergency physical first aid (PFA) training course among 1942 students in Year 10 at four secondary schools in Australia.

*Mental health first aid* is defined as the support provided to an individual who is developing a mental health problem, experiencing a worsening of an existing problem, or in a mental health crisis. Support is given until appropriate help is received or the crisis resolves [12]. Mental health first aid skills have been taught in Mental Health First Aid Australia (MHFA) training programs, first developed by Kitchener and Jorm in 2000 [13]. Two recent meta-analyses of MHFA courses found that training designed for adults decreased the recipients’ stigmatising attitudes towards individuals with mental illness, improved their mental health literacy, confidence in providing first aid, quality of first aid intentions, and beliefs about appropriate treatment, for up to 6-months following the training [14, 15].

teen Mental Health First Aid (tMHFA) is a program designed to teach adolescents to better help peers with MHPs or crises [16–18]. The senior version of the tMHFA course is comprised of three 75-min sessions and is targeted at teenagers in years 10 to 12 of secondary school in the Australian education system [18]. As previously described [17], tMHFA aims to improve mental health literacy, increase the quality of first aid actions provided to peers—including those at risk of suicide—and decrease stigmatising attitudes towards mental illness. The course was developed from research evidence on effective, age-appropriate first aid strategies and importantly, emphasises when and how to enlist the help of an appropriate adult [17].

An initial evaluation of tMHFA involved an uncontrolled pilot trial with 988 students across four Australian schools. Student survey data collected at baseline, one week post-training and three months later showed significant increases in students’ confidence in providing first aid to a friend, mental health literacy, and help seeking intentions, as well as decreased stigma and psychological distress following the training [17]. A subsequent cluster crossover randomized controlled trial [19] was then conducted comparing tMHFA to a matched PFA training program. Significant group-by-time interactions with medium effect sizes favouring tMHFA were found on quality of first aid intentions, on confidence supporting a peer, and number of adults rated as helpful. Greater reductions in stigmatising beliefs, and ‘unhelpful first aid intentions’, were also reported. The current paper builds on those pre-post results by reporting on student outcomes at 12-month follow-up in the same CRXO trial [20].

It was hypothesised that, at follow-up as compared to baseline, adolescents who received tMHFA would report greater increases in the quality of first aid intentions and behaviours, confidence, help-seeking intentions and beliefs about the helpfulness of adults—as well as greater decreases in stigmatising attitudes and unhelpful first aid intentions and behaviours—than students who received PFA training.

## Methods

The descriptions below follow the CONSORT 2010 statement and extension for cluster randomised trials [21].

### Aim

The CRXO trial aimed to examine the efficacy of the tMHFA intervention in improving students’ mental health first aid towards peers, increasing mental health literacy, and reducing stigmatising attitudes, as compared to a matched comparator PFA intervention. The current paper aimed to evaluate students’ longer-term outcomes by analysing data collected one year after participation in either the tMHFA or a matched PFA training program.

### Trial design

CRXO is a refinement of the matched-pair cluster design; in CRXO trials, all clusters (schools) receive all interventions with the sequence of administration being randomly assigned. In the current study, two schools were enrolled as a matched pair, based on similar scores on the Index of Socio-educational Advantage (ICSEA) and the size of their Year 10 cohort. It was a condition of participation that schools agreed to have all students in their Year 10 cohort eligible to receive first aid training and to participate in evaluation surveys. Schools were randomised to receive either intervention sequence one (tMHFA provided in Year 1, followed by PFA provided in Year 2), or sequence two (PFA provided in Year 1, followed by tMHFA provided in Year 2). Two matched pairs (four schools in total) were randomised to receive either tMHFA or PFA in the first year for their entire Year 10 cohort. The trial was conducted over four years from 2014 to 2017. In 2014, the first pair of schools received their first intervention (one school received tMHFA and the other PFA). In 2015, these schools received the opposite intervention for their new Year 10 cohort, while their previous cohort completed the 12-month follow-up survey. In addition, a new pair of schools joined the trial and received their first year of interventions. In 2016, the second Year 10 cohorts in the first pair of schools completed their 12-month follow-up surveys, while the second pair of schools received their opposite intervention. In 2017, the second cohort of students in the second pair of schools completed their follow-up surveys. This design allows counterbalancing across schools, whilst also controlling for within-school variance, by using intervention crossover in subsequent waves. Individual school characteristics are shown in Table 1.

**Table 1:** Participant characteristics by school and intervention

	**tMHFA**^**a**^	**PFA**^**b**^	**Combined**
**School 1** (ICSEA = 1031)			
Eligible at assignment	159	170	329
Sample at baseline (n)^c^	116	115	231
Sample at 1 year follow-up (n)	61	61	122
Age (M, SD)	15.92, 0.52	16.03, 0.52	15.97, 0.52
Gender (% female)	36.21	38.26	37.23
English First Language (% yes)	74.14	74.78	74.46
**School 2** (ICSEA = 1050)			
Eligible at assignment	230	230	460
Sample at baseline (n)	200	209	409
Sample at 1 year follow-up (n)	116	133	249
Age (M, SD)	15.65, 0.39	16.06, 0.40	15.86, 0.45
Gender (% female)	48.00	45.45	46.70
English First Language (% yes)	84.5	87.08	85.82
**School 3** (ICSEA 1091)			
Eligible at assignment	300	280	580
Sample at baseline (n)	231	233	464
Sample at 1 year follow-up (n)	101	114	215
Age (M, SD)	15.84, 0.42	15.76, 0.44	15.80, 0.43
Gender (% female)	38.53	45.92	42.24
English First Language (% yes)	85.28	83.26	84.27
**School 4** (ICSEA 1097)			
Eligible at assignment	300	273	573
Sample at baseline (n)	261	240	501
Sample at 1 year follow-up (n)	185	120	305
Age (M, SD)	15.89, 0.62	15.90, 0.64	15.89, 0.63
Gender (% female)	49.04	48.75	48.90
English First Language (% yes)	52.11	47.08	49.7
**Total Sample**			
Eligible at assignment	989	953	1942
Sample at baseline (n)	808	797	1605
Sample at 1 year follow-up (n)	463	428	891^d^
Age (M, SD)	15.82, 0.51	15.92, 0.52	15.87, 0.52**
Gender (% female)	43.94%	45.55%	44.74%
English First Language (% yes)	72.77%	72.15%	72.46%

^a^All students in Year 10 received teen Mental Health First Aid training (tMHFA)

^b^All students in Year 10 received Physical First Aid training (PFA)

^c^All students who completed student assent, age and gender, and at least one item on the John vignette were included in the analytic sample

^d^Analyses were conducted according to the intention to treat principle; all participants with missing data at follow-up were included in analyses (*n* = 1605) as well as an additional three students who gave data at follow-up only (*n* = 1608)

^**^Significant group difference found at the *p* ≤ .001 level

### Participants

To be eligible, schools needed to be Government funded (rather than independent/private), agree to two consecutive cohorts of Year 10 students undertaking three survey sessions (baseline, post-training and 12-month follow-up) and three training sessions (once per week over three weeks) in regular class time, and agree to withhold any overlapping mental health classroom curriculum or programs, until the completion of the research. Students were eligible to participate in the evaluation surveys if they had parental consent and provided assent at the beginning of each survey. Students with a known mental health problem, previous experience of mental illness, suicide behaviour or bereavement, were encouraged to speak to their mental health professional, school counsellor and/or parents before deciding whether to participate. Passive parental consent was used. The research team provided parent, teacher and student information sessions, and electronic and hardcopy information statements, three weeks prior to baseline measures. Parents could opt their child out of the training or the evaluation by returning a signed form to the participating school. To protect the identity of these families, no data were gathered on non-consenters.

### Interventions

Both interventions consisted of three 75-min classroom sessions, presented by trained instructors external to the host school, according to a manualised curriculum. In each intervention, students were provided with a specific program booklet and completion certificate. Training was normally completed within three weeks (one session per week).

#### teen Mental Health First Aid

A detailed explanation of the tMHFA program, including a detailed description of the training curriculum and the frameworks that informed its development, has been provided elsewhere [17], but relevant details are noted here. Session 1 covered information about mental illness, prevalence, burden and the importance of early intervention. Session 2 covered the tMHFA action plan and how to help a friend experiencing a mental health crisis. Session 3 provided further detail on the action plan, appropriate professional help seeking and how to help a friend who is developing a mental health problem. Training for students involved a PowerPoint presentation, videos, role-plays, group discussion, small group and workbook activities, all manualised and delivered by trained instructors who completed at least 5.5 days of training [17, 18].

Because a core message of the tMHFA training is to seek assistance from a trusted and reliable adult when a peer is experiencing a mental health problem, the 14-h Youth MHFA course [22] was also offered to staff and parents (separately) at participating schools. This course is for adults living or working with young people and ensured that adults who were likely to be called upon to assist adolescents as a result of tMHFA were confident in providing support and could facilitate appropriate referral pathways.

#### Physical first aid

PFA training for students was adapted from the Basic First Aid training program for adults provided by Red Cross. Session 1 covered the DRSABCD action plan, CPR and use of an automated external defibrillator. Session 2 covered basic first aid for common accidents and injuries in adolescents, including sprains, strains, wound care, fractures and dislocations, concussions and asthma. Session 3 covered anaphylaxis, poisons, exposure to heat and cold, diabetes and seizure.

Program delivery involved instructor introductions of topic content, role-plays using mannequins, bandages and splints, and group discussions. PFA instructors underwent a minimum of 3 days training in first aid, plus an additional certificate-level course in Workplace Training and Assessment (8-week full-time equivalent).

### Outcome measures

The survey, which was modified from previous national mental health literacy surveys with youth [23] and evaluations of tMHFA [17], measured *mental health first aid* (helpful and unhelpful intentions, confidence in providing help, quality of mental health first aid behaviours provided to a peer and quality of mental health first aid behaviours received from a peer in the last 12-months), *mental health literacy* (beliefs about helpfulness of adult sources of help, help-seeking intentions), and *stigmatising beliefs* (social distance, weak-not-sick, dangerous/unpredictable and would not tell anyone). A measure of generalised psychological distress (the Kessler six item; K6), of correct recognition of the diagnostic label in the vignette, and of experiences with physical first aid in the past year, were also administered at baseline and 12-month follow-up, but results are published elsewhere [19, 24].

The survey presented two vignettes: one (John) depicting an adolescent with suicidal ideation and symptoms matching DSM-5 and ICD-10 criteria for a depressive disorder [25, 26], and another (Jeanie) with symptoms matching criteria for social anxiety/phobia [25, 26]. Vignettes are provided in Supplementary Document 1. A description of all measures, including reliability coefficient omega (ω), is provided in Table 2 [27].

**Table 2:** Measures used at baseline and one year follow-up to assess student outcomes

Outcome	Survey measure	Response	Example	Scores range and reliability statistics
**Measures of Mental Health First Aid**				
Quality of first aid intentions	*If John/Jeanie was a friend I would…*	Choose any of 13 possibilities: 6 consistent with Action Plan 6 discordant with Action Plan *Other (please specify)*	Helpful—*Tell John I have noticed something seems wrong, and I want to make sure he is okay* Harmful/unhelpful—*Ignore Jeanie because she is attention-seeking*	Helpful intentions subscale, total score on 6 items: 0 to 6 John ω = .84 Jeanie ω = .85 Unhelpful intentions subscale, total score on 6 items 0 to 6 John ω = .76 Jeanie ω = .78
Confidence	*If John/Jeanie was a friend, how confident would you feel in helping him/her*	5-point Likert scale 1 = Not at all 5 = Extremely		Test–retest for PFA students (apx 4 weeks apart) John *r* = .52 Jeanie *r* = .48
Quality of first aid behaviours (among those reporting contact with a peer with a mental health problem/crisis)	*In the last 12 months have you had contact with anyone about your age (i.e. between* *13–18 years old) who you thought might have a mental health problem or has experienced a mental health crisis?*	Yes, No, Not sure, I do not want to answer If yes/not sure – *Please tell us how many people about your age you had contact with who you* *thought might have a mental health problem or experienced a mental health crisis; did you offer any help?; What did you do to help the person?* Choose any of 12 possibilities: 6 consistent with Action Plan 6 discordant with Action Plan *Other (please specify)*	Helpful – *Invited them to hang out and do something fun with me* Unhelpful – *Avoided talking about suicide because it might put the idea in their head*	Helpful behaviours subscale, total score on 6 items: 0 to 6 ω = .73 Unhelpful behaviours subscale, total score on 6 items 0 to 6 ω = .64
Quality of first aid received from a peer (among those self-reporting a mental health problem/crisis)	*In the last 12 m has anyone tried to support or assist you with this mental health problem or crisis?*	Yes, No, Not sure If yes/not sure—*Who provided support or assistance for the problem?* [friend, parent, other family member, health professional, teacher, other] If friend – *What did your friend do to help you?* Choose any of 10 possibilities: 6 consistent with Action Plan 4 discordant with Action Plan Other (please specify)	Helpful – *Told me they noticed something seems wrong and wanted to make sure I was ok* Harmful/Unhelpful – *Told me what to do to fix my problems*	Helpful behaviours received subscale, total score on 6 items: 0 to 6 ω = .71 Unhelpful behaviours received subscale, total score on 4 items 0 to 4 ω = .67
**Measures of Mental Health Literacy**				
Beliefs about helpfulness of adult help	*Which of the following people do you think would be helpful, harmful, or neither for John/Jeanie’s problem?*	Helpful, Neither, Harmful	*Counsellor* *General Practitioner (GP) or family doctor* *Parent* *Teacher* *School Counsellor/Welfare coordinator* *Minister or Priest*	Total number of adults endorsed as helpful: 0 to 6 Test–retest for PFA students (apx 4 weeks apart) John *r* = .55 Jeanie *r* = .58
Help seeking intentions	*If I had a problem right now like John’s/Jeanie’s I would…*		*Talk to a friend about it* *Improve my diet*	Total score on 5 items: 0 to 5 John ω = .76 Jeanie ω = .72
**Measures of Stigma**				
Social Distance	*Would you be happy to:*	4-point Likert scale 1 = Yes definitely 4 = Definitely not	*Develop a close friendship with John/Jeanie* *Go out with John/Jeanie on the weekend*	Total score on 5 items: 5 to 20 John ω = .91 Jeanie ω = .94
Weak-not-sick	*Please indicate how strongly you personally agree or disagree with each statement*	5-point Likert scale 1 = Strongly disagree 5 = Strongly agree	*A problem like John/Jeanie’s is a sign of personal weakness* *John/Jeanie’s problem is a real medical illness* *People with a problem like John/Jeanie’s could snap out of it if they wanted* *It is best to avoid people with a problem like John/Jeanie’s so you don’t develop this problem*	Mean score of 4 items John ω = .77 Jeanie ω = .78
Dangerous/Unpredictable	*Please indicate how strongly you personally agree or disagree with each statement*	5-point Likert scale 1 = Strongly disagree 5 = Strongly agree	*People with a problem like John’s/Jeanie’s are dangerous* *People with a problem like John/Jeanie’s are unpredictable* *It is best to avoid people with a problem like John/Jeanie’s so you don’t develop this problem*	Mean score of 3 items John ω = .59 Jeanie ω = .77
Would not tell anyone	*Please indicate how strongly you personally agree or disagree with each statement*	5-point Likert scale 1 = Strongly disagree 5 = Strongly agree	*If I had a problem like John’s/Jeanie’s I would not tell anyone*	Test–retest for PFA students John *r* = .54 Jeanie *r* = .50

ω = Revelle’s omega total for total scores and subscales. Omega is considered acceptable when above 0.70

*r* = was calculated based on the control condition measures taken at baseline and post-training which occurred approximately 4 weeks apart. Given that an intervention was provided in between the measurement occasions, this may have led to lower reliability estimates than would be reported by a training-naive sample. Other test–retest reliability data on these instruments has not been previously developed, as this was the first implementation of the John and Jeanie vignettes in a tMHFA-naive sample

#### Measures of mental health first aid

The primary outcome for this study was *quality of mental health first aid intentions*. Participants were asked to endorse any of 12 possible actions towards John, then again towards Jeanie. The possible actions were designed to be consistent with the tMHFA action plan (i.e., *helpful intentions*) or contrary to the plan as distractors (i.e., *unhelpful intentions*; see Table 2 for examples). For the *helpful intentions* subscale, a simple count of the number of actions endorsed was used. For the *unhelpful intentions* subscale, scores were non-normally distributed with heavy skew, as these actions were rarely endorsed. Accordingly, scores were dichotomised at 0/1–6 and mixed effects logistic regression models run. The quality of intentions measure was chosen because research shows that intention ratings correlate strongly with future behaviours [28] and because the quality of young people’s mental health first aid intentions has been shown to predict subsequent helping actions two years later [29]. Further, all participants could report on their first aid intentions, allowing adequate power to detect group x time interactions with the full sample; changes in mental health first aid behaviours provided to a peer could only be analysed on the smaller subset of respondents who reported having contact with a peer experiencing a mental health problem or crisis.

*Confidence* in providing first aid to a peer was measured with a single item on a 5-point Likert scale, with higher scores indicating higher confidence [17, 30].

*Quality of mental health first aid behaviours* was examined using the First Aid Experiences Questionnaire [17]. This was presented last in the survey, to avoid priming responses to the vignette-based measures. Definitions were first provided to students, in accordance with the teachings of the tMHFA program; a mental health problem was defined as “a major change in a person’s normal way of thinking, feeling or behaving, which interferes with the person’s ability to get on with life, and does not go away quickly or lasts longer than normal emotions or reactions would be expected to”. The survey stated that this might involve a diagnosed mental illness, a worsening of mental health, an undiagnosed problem, or a drug or alcohol problem. A mental health crisis was defined as when “a person is at increased risk of harm to themselves or to others”. The survey stated that crisis situations might include having thoughts of suicide, engaging in self-injury, being very intoxicated with alcohol or other drugs, or experiencing bullying or abuse. Students’ first aid experiences were assessed at baseline and follow-up by asking if in the last 12-months they had contact with anyone who they thought might have a mental health problem or experienced a mental health crisis. If they reported having contact, the student was asked how many people they had contact with, and whether they had offered any help. If students answered ‘yes’ or ‘not sure’ to offering help, they were then asked “What did you do to help the person?” and were presented with 12-response options (these were the same as the options shown in the quality of first aid intentions measure). Six options were concordant with the tMHFA action plan (i.e., *helpful behaviours*), and six were discordant (i.e. *unhelpful* behaviours); students could select as many as applied. The use of forced-choice responses was an adaptation from previous research, which found that when asked to provide free-text descriptions of first aid behaviours students provided such scant detail, then further truncated responses over time, that quantitative analyses could not be reliably conducted [17]. Questions about experiences providing and receiving first aid for medical emergencies or physical injuries were also asked and these results have been published elsewhere [24]. *Quality of first aid behaviours* was measured by calculating the mean number of helpful and unhelpful responses, when taking into account having had the opportunity to provide mental health first aid (i.e. having reported being in contacted with someone their age who was experiencing a mental health problem or crisis). For the *unhelpful behaviours* subscale, scores were again non-normally distributed, and therefore dichotomised at 0/1–6.

At baseline and follow-up, students were also asked if they had experienced a mental health problem or crisis themselves in the last 12-months. Students were instructed to report any mental health problem they thought they might have experienced, whether or not this was formally diagnosed by a health professional. For those who said ‘yes’ or ‘not sure’, further questions were asked, including whether in the last 12-months someone had tried to support them with their problem or crisis, and from whom this was received (friend, parent, other family member, health professional or teacher). Where a friend was reported, students were asked what the friend did to help. To respond, students could select from 10 responses; six were helpful actions and four unhelpful/harmful actions, as per the provision of quality MHFA intentions (the options ‘did nothing’ and ‘avoided talking about suicide because it might have put the idea in their head’ were omitted from the original 12-item list). *Quality of first aid received* was measured by calculating the mean number of *helpful* and *unhelpful behaviours received* from a friend, taking into account the number of students with the opportunity to receive mental health first aid (i.e. having reported experiencing a mental health problem or crisis themselves) within the last 12-months. Again, due to non-normal distribution, the *unhelpful behaviours received* variable was dichotomised.

#### Measures of mental health literacy

Beliefs about sources of adult help were assessed by asking participants to rate the helpfulness of a range of individuals who have a role in student wellbeing (e.g., teacher, parent). Although beliefs about self-help or social support have also been a feature of other mental health literacy research [31, 32], the current study only analysed data on adult sources of help because the need to engage adult help is a key message of the tMHFA training. The number of adults that were endorsed as *helpful* was counted at baseline and again at follow-up.

To examine help-seeking intentions, students were asked to respond to the question *If I had a problem right now like John’s, I would…* with 10 response options provided. Students were asked to endorse as many options as they liked. A 5-item unidimensional *help-seeking intentions* scale was formed from three items addressing support from others and two items on avoiding help, which were reverse scored. Scores for this scale were calculated by summing the number of items endorsed. The remaining items were self-help strategies, which were not an explicit focus of tMHFA and included as distractor items.

#### Measures of stigma

Measures of stigmatising beliefs included the Social Distance Scale (SDS) [33] and the Depression Stigma Scale (DSS) [34, 35], modified for use with the John/Jeanie vignettes [17] and evaluated as four distinct factors: *social distance*, *weak-not-sick*, *dangerous/unpredictable* and *would not tell anyone* [36]. The first three factors were scales used as measures of personal stigma (i.e. the negative attitudes held about others with a mental health problem) while the last was a single item measuring self-stigma (i.e. the internalisation of negative attitudes others’ hold about mental health problems, directed towards the self).

#### Covariates

Students were also asked demographic questions and completed the K6 [37]. Students were asked to indicate their age in years and the number of months since their last birthday (0–11). To indicate gender, students were asked to select from the options ‘male’, ‘female’, ‘identify with another term’. Students were also asked if English was their first language/the language they spoke at home (‘yes’ or ‘no’). Gender and age were significant predictors of data missingness, while gender and K6 scores significantly predicted the likelihood of offering and receiving help over the past 12-months. Language was not significantly associated with outcome measures and therefore not entered into analyses.

### Statistical analysis

The data were analysed with mixed models for continuous and binary outcome variables, with group-by-measurement-occasion interactions. This method takes into account the data’s hierarchical structure (i.e. the correlation of measurement occasions within students and within clusters). These maximum likelihood-based methods are able to produce unbiased estimates when a proportion of the participants withdraw before the completion of the study, based on the reasonable assumption that these data are missing at random.

With the cluster-crossover design of four schools and two periods (eight x year 10 cohorts), models included a random effect for the school-year cohort clusters, and fixed effects for school, year, gender and age. This adjusts for the correlation of student responses within school-year cohort clusters. Gender and age were included as fixed effects in order to help meet the missing at random assumption, given they were associated with missingness.

School year cluster intraclass correlation coefficient (ICC) indicates the proportion of variability in the outcome attributable to school-year clusters. Where there were no baseline imbalances, effect sizes (Cohen’s *d*) were calculated by dividing the difference between the two group means at 12-month follow-up by their pooled standard deviation. With baseline imbalances, Cohen’s *d* was calculated by dividing the mean change score in each condition by the pooled standard deviation at follow-up. Analyses were performed in Stata 14 between 2018–2021.

### Statistical Power

It was conservatively estimated that there would be 100 Year 10 students per school, with a 50% consent + assent rate. Across four schools and eight clusters, this would give 400 adolescents (200 per intervention). The estimated intra-class correlation for students (ICC = 0.003) at the school cluster level was based on findings from previous research [17] and not included in the power calculations due to the likely small design effect and the counterbalancing of schools. With an assumed 0.70 correlation between pre-post measurements, the study would have a 0.80 power to detect small (*d* = 0.17) group-by-measurement occasion differences at α = 0.05.

### Implementation

The research team emailed a request to all accredited Youth MHFA instructors in the Melbourne area asking for an introduction to any secondary schools with an expressed interest in receiving training for staff and students. Two schools agreed to participate through this mechanism. A further two schools were selected to form matching pairs on Index of Socio-Educational Advantage (ICSEA; *M* = 1000, *SD* = 100) [38] and Year 10 cohort size in the first year. Schools were required to be within 1.5*SD* on ICSEA and within 100 students in size. These latter two schools did not have previous MHFA training and were ‘cold called’. The resulting four schools were enrolled in the trial upon completion of a research agreement signed by the principal. A random sequence generator (SPSS) was used by the trial manager to generate the treatment sequence for the two pairs of schools, with the research assistant assigning the first school enrolled to sequence one, the second school enrolled to sequence two, and the remaining schools assigned according to the opposite sequence of their matched pair. Although randomisation was at the cluster level, the primary outcome was analysed at the individual level, as the aim of this research was to understand the impact of first aid training on students’ supportive intentions towards their peers, rather than impact on the school community. Research assistants, instructors providing training and students could not be blinded to intervention. However, research staff coding and analysing data were blinded to measurement occasion, school identity and condition. All survey and intervention sessions were conducted between April 2014 and August 2017. Although the paid provider of the PFA sessions changed after year 1, no changes to the content presented or research methods occurred after trial commencement.

## Results

**Fig. 1:**
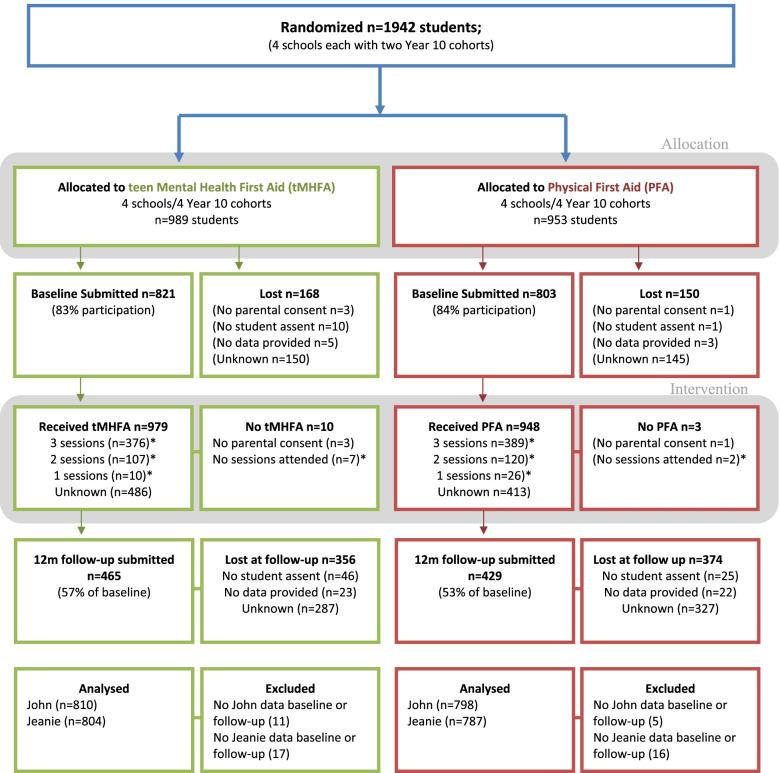
CONSORT Participant flow diagram

The flow of participants throughout the trial is depicted in Fig. 1.

### Mental health first aid

Table 3 outlines results for continuous and categorical mental health first aid outcomes at 12-month follow up as compared to baseline, for students undertaking PFA and tMHFA.

**Table 3:** Outcomes on measures of mental health first aid intentions, confidence and behaviours

**MHFA outcomes**	**Baseline**				**1-year follow up**								
	**PFA**^**a**^		**tMHFA**^**b**^		**PFA**^**c**^		**tMHFA**^**d**^						
	**M**	**SD**	**M**	**SD**	**M**	**SD**	**M**	**SD**	**M diff**	**95% CI**	***p***	***d***^**e**^	***ICC***^**f**^
***Quality of first aid intentions***^**g**^													
Helpful intentions – John	3.76	1.58	3.77	1.49	3.62	1.51	4.06	1.67	0.44	0.25–0.64	** < .001**	0.28	.004
Unhelpful intentions – John^h^	68.1%		68.3%		66.2%		53.8%		OR 0.42	0.27–0.65	** < .001**^**a**^		.000
Helpful intentions – Jeanie	3.22	1.58	3.3	1.53	3.32	1.51	3.57	1.62	0.2	-0.01–0.4	0.056	0.16	.003
Unhelpful intentions – Jeanie^h^	60.3%		57.5%		53.3%		49.1%		OR 0.83	0.54–1.29	.419^a^		.001
***Confidence in helping***													
John	3.63	0.99	3.54	0.97	3.34	1.02	3.58	0.94	0.33	0.21–0.46	** < .001**	0.24	.004
Jeanie	3.7	0.99	3.69	1.01	3.54	0.99	3.71	0.92	0.15	0.02–0.28	**0.021**	0.18	.008
***Quality of first aid provided to a peer***													
Helpful behaviours provided^i^	3.08	1.67	3.09	1.72	3.11	1.64	3.39	1.64	0.26	-0.10–0.63	.159	0.17	0.000
Unhelpful behaviours provided^j^	60%		54.0%		49%		35%		OR 0.62	0.31–1.22	.163		0.000
***Quality of first aid received from a peer***													
Helpful behaviours received^k^	2.99	1.57	2.86	1.46	3.13	1.52	2.54	1.36	-0.53	-1.12–0.06	.079	-.4	0.070
Unhelpful behaviours received^l^	49%		48%		47.2%		34.4%		OR 0.34	0.10–1.20	.093		0.000

All mixed models were adjusted for school, year, gender, age (and K6 scores for *Quality of first aid provided to/received from a peer*). Boldface indicates statistical significance

^a^For John vignette depicting peer with symptoms of depression & suicide risk *n* = 791; for Jeanie vignette depicting peer with symptoms of social anxiety *n* = 769

^b^For John vignette *n* = 807; for Jeanie vignette *n* = 792

^c^For John vignette *n* = 429; for Jeanie vignette *n* = 409

^d^For John vignette *n* = 465; for Jeanie vignette *n* = 446

^e^Large effect size Cohen’s *d* = 0.8, medium effect size d = 0.5, small effect size d = 0.2

^f^Period-cluster intraclass correlation coefficients (ICC) indicate the proportion of variability in the outcome attributable across clusters

^g^This measure was the primary outcome

^h^Scores were non-normally distributed (rarely endorsed), so were dichotomised at 0/1–6; values shown are % scoring 1 or above and mixed effects logistic regression models

^i^Includes those who reported offering help at either timepoint; *n* = 483 at baseline, *n* = 136 at follow-up, *n* = 215 at both points, *n* = 834 total

^j^Scores were non-normally distributed with heavy skew (rarely endorsed), so were dichotomised at 0/1–6; values shown are % scoring 1 or above and mixed effects logistic regression models based on scores from *n* = 834

^k^Includes only those who reported experiencing a mental health problem at baseline and 12 m follow-up and receiving help from a peer; *n* = 309

^l^Scores were non-normally distributed with heavy skew (rarely endorsed), so were dichotomised at 0/1–6; values shown are % scoring 1 or above and mixed effects logistic regression models based on scores from *n* = 309

#### Quality of first aid intentions

On the *helpful first aid intentions* subscale for the John vignette, a significant group x time interaction was found. From baseline to follow-up, students receiving tMHFA endorsed significantly more helpful first aid intentions towards John, than students receiving PFA. For Jeanie, although the number of helpful intentions endorsed was slightly higher at follow up than at baseline among the tMHFA students, this did not quite reach statistical significance (*p* = 0.056) when compared to scores over time reported by those receiving PFA.

On the *unhelpful first aid intentions* subscale for the John vignette, a significant group x time interaction was also found. The proportion of students receiving tMHFA who endorsed at least one unhelpful first aid action towards John, reduced from baseline to follow-up. Although the proportion of students receiving PFA who endorsed at least one unhelpful action towards John also reduced over time, this was much smaller. On the *unhelpful intentions* subscale for the Jeanie vignette, no significant group x time interaction was found. However, the endorsement rate was consistently low for Jeanie and perhaps this led to a floor effect.

#### Confidence

Significant group x time interactions were found for students’ confidence in helping both John and Jeanie. Twelve months after receiving training, tMHFA students reported higher levels of confidence in helping a peer, than students receiving PFA did, for both vignettes.

#### Quality of first aid behaviours provided to a peer

At baseline 430 (56%) of PFA students and 430 (55%) of tMHFA students reported having contact with a peer experiencing a mental health problem or crisis. Of those, 335 (78%) of PFA and 363 (84%) of tMHFA students reported offering the peer some help. This difference was statistically significant (OR = 1.93, CI95% = 1.10–3.39, *p* = 0.023), suggesting an imbalance upon randomisation had occurred.

At follow up, 201 (50%) PFA students and 199 (45%) tMHFA students reported having contact, and of those, 176 (88%) PFA and 175 (88%) tMHFA students reported offering help. There were 104 PFA students and 111 tMHFA students who reported offering help at both time points. There was no significant group x time interaction in the odds of offering help to a peer with a mental health problem. In addition to being in the tMHFA group, being female and having a higher K6 score were also significant predictors of offering help at baseline. As a result, these variables were accounted for in the multilevel models, as presented in Table 3.

Any student who reported offering help at either time point was included in the *quality of help provided to a peer* measure and their scores subject to multilevel analysis. There was no significant group x time interaction, however, at follow up the mean score on the *helpful behaviours* subscale had increased by 0.3 points among tMHFA students, but by 0.03 points among PFA students. This difference was associated with a small effect size, though not statistically significant. Both females and those with higher K6 scores were more likely to report helpful behaviours.

The proportion of students endorsing at least one unhelpful behaviour at baseline was 60% for PFA students and 54% for tMHFA students. At 12-month follow up, this had changed to 49% for PFA students and 35% for tMHFA students. The reduction in unhelpful behaviours was statistically significant for both groups. Although tMHFA students showed slightly greater reductions than PFA students, there was no significant group x time interaction. Females were less likely than males to report at least one unhelpful behaviour.

#### Quality of first aid behaviours received from a peer

At baseline, 15.6% (*n* = 119) PFA students and 17.5% (*n* = 137) tMHFA students self-reported a mental health problem or crisis in the last 12-months. Of those, 74% of PFA (104) and 73.5% of tMHFA (125) students reported having received help from a peer. At follow-up 18% (73) PFA students and 15.4% (67) of tMHFA students reported a mental health problem or crisis in the previous 12 months. Of those, 79% of PFA students (72) and 63.5% of tMHFA students (61) reported having received help from a peer. There was no significant group x time interaction for frequency of reporting having received help from a peer. There were 26 PFA students and 27 tMHFA students who reported having had a mental health problem or crisis and reported having received help from a peer at both time points. Consistent with intention-to-treat principles, all students who reported receiving help at any time were subject to multilevel analysis on the *quality of help received from a peer* measure, as presented in Table 3 (*n* = 150 PFA, *n* = 159 tMHFA). Females and those with higher K6 scores were significantly more likely to report having received help from a peer and these variables were therefore included as fixed effects in the multilevel models.

The mean number of *helpful behaviours received* from peers, as reported at both baseline and follow-up, was around 3 (of a possible 6) and did not show any significant changes over time, nor significant group x time interaction. Those reporting higher K6 scores, older age and female gender were significantly more likely to report having received helpful behaviour from a peer, regardless of group allocation.

The proportion of students who reported one or more *unhelpful behaviours received* also showed no significant changes over time, across groups, or interaction effects. Those reporting higher K6 scores were significantly more likely to report having received an unhelpful behaviour from a peer and this was therefore accounted for in the multilevel model.

### Mental health literacy

Table 4 outlines results for mental health literacy outcomes. Significant group x time interactions were found for the number of adults that students rated as helpful for both John and Jeanie. One year after receiving training, students receiving tMHFA listed more adults as helpful than students receiving PFA.

**Table 4:** Outcomes for measures of mental health literacy

**Mental health literacy outcomes**	**Baseline**				**1-year follow up**								
	**PFA**^**a**^		**tMHFA**^**b**^		**PFA**^**c**^		**tMHFA**^**d**^						
	**M**	**SD**	**M**	**SD**	**M**	**SD**	**M**	**SD**	**M diff**	**95% CI**	***p***	***d***^**e**^	***ICC***^**f**^
***Beliefs about adult help***													
John^g^	3.43	1.72	3.45	1.62	3.47	1.64	3.81	1.57	0.34	0.13–0.54	**0.002**	0.21	0.012
Jeanie^g^	2.79	1.93	2.71	1.83	3.09	1.76	3.38	1.74	0.38	0.14–0.62	**0.002**	0.25	0.003
***Help seeking intentions***													
John	2.97	1.34	2.94	1.34	2.90	1.40	3.16	1.35	0.27	0.10–0.44	**.002**	0.19	0.000
Jeanie	2.95	1.30	2.96	1.26	2.85	1.35	3.21	1.28	0.31	0.14–0.48	** < .001**	0.27	0.003

All mixed models were adjusted for school, year, gender, age and K6 scores. Boldface indicates statistical significance

^a^For John vignette depicting peer with symptoms of depression & suicide risk *n* = 791; for Jeanie vignette depicting peer with symptoms of social anxiety *n* = 769

^b^For John vignette *n* = 807; for Jeanie vignette *n* = 792

^c^For John vignette *n* = 429; for Jeanie vignette *n* = 409

^d^For John vignette *n* = 465; for Jeanie vignette *n* = 446

^e^Large effect size Cohen’s d = 0.8, medium effect size d = 0.5, small effect size d = 0.2

^f^Period-cluster intraclass correlation coefficients (ICC) indicate the proportion of variability in the outcome attributable across clusters

^g^Scored when the following adults were rated as ‘Helpful’ (as opposed to Neither or Harmful): Counsellor, GP or family doctor, Minister or priest, Psychologist, School welfare coordinator/School counsellor, Teacher (0 to 6)

Significant group x time interactions were also found for student ratings on the help-seeking intentions measure, in response to both vignettes. One year after training, tMHFA students were more willing to seek support from others if they were to experience a problem like John or Jeanie’s, while the willingness of students receiving PFA had decreased. Changes over time for both vignettes were associated with small effect sizes, though effects were slightly larger in response to the Jeanie vignette.

### Stigma

Table 5 reports the outcomes on measures of stigma. For three measures (social distance, weak-not-sick, and dangerous/unpredictable) no significant group x time interactions were found. For the *would not tell anyone* measure, tMHFA students reported greater reductions over time, in response to both the John and Jeanie vignette, than students who received PFA.

**Table 5:** Outcomes for measures of stigma

**Stigma outcomes**^**a**^	**Baseline**					**1-year follow up**									
	**PFA**^**b**^			**tMHFA**^**c**^		**PFA**^**d**^			**tMHFA**^**e**^						
	**M**	**SD**	**M**		**SD**	**M**	**SD**	**M**		**SD**	**M diff**	**95% CI**	***p***	***d***^**f**^	***ICC***^**g**^
***Social distance***															
John	1.98	0.64	1.96		0.64	1.97	0.64	1.87		0.62	-0.07	-0.14–0.00	0.065	0.15	.014
Jeanie	1.78	0.65	1.71		0.64	1.74	0.63	1.64		0.63	-0.01	-0.08–0.07	0.879	0	.000
***Weak-not-sick***^**h**^															
John	2.09	0.83	1.97		0.81	1.95	0.86	1.83		0.83	-0.03	-0.12–0.05	0.422	0.06^b^	.029
Jeanie	2.25	0.85	2.15		0.87	2.07	0.87	1.93		0.88	-0.07	-0.16–0.02	0.136	0.09^b^	.043
***Dangerous/Unpredictable***^**h**^															
John	2.51	0.75	2.39		0.75	2.35	0.79	2.15		0.78	-0.09	-0.19–0.01	0.067	0.15^b^	.025
Jeanie	2.01	0.78	1.86		0.76	1.93	0.84	1.74		0.81	-0.05	-0.15–0.05	0.368	0.06^b^	.008
***Would not tell anyone***^**h**^															
John	2.53	1.08	2.41		1.10	2.61	1.07	2.35		0.99	-0.17	-0.30—-0.03	**0.016**	0.17^b^	.005
Jeanie	2.46	1.09	2.37		1.08	2.52	1.08	2.20		1.03	-0.24	-0.38—-0.09	**0.002**	0.24^b^	.000

All mixed models were adjusted for school, year, gender, age and K6 scores. Boldface indicates statistical significance

^a^For all stigma items a reduction in scores was a positive outcome, indicating lower stigmatising beliefs

^b^For John vignette depicting peer with symptoms of depression & suicide risk *n* = 791; for Jeanie vignette depicting peer with symptoms of social anxiety *n* = 769

^c^For John vignette *n* = 807; for Jeanie vignette *n* = 792

^d^For John vignette *n* = 429; for Jeanie vignette *n* = 409

^e^For John vignette *n* = 465; for Jeanie vignette *n* = 446

^f^Large effect size Cohen’s d = 0.8, medium effect size d = 0.5, small effect size d = 0.2

^g^Period-cluster intraclass correlation coefficients (ICC) indicate the proportion of variability in the outcome attributable across clusters

^h^Calculated on change over time rather than difference at follow-up due to baseline imbalances

## Discussion

This study aimed to establish the longer-term outcomes of the tMHFA program by examining data collected one year after training. Across all domains tested (mental health first aid, mental health literacy, and stigmatising attitudes) students receiving tMHFA reported significantly better improvements on at least one measure, compared to PFA students. Results suggest that the tMHFA program had positive impacts on adolescents’ mental health knowledge, attitudes and behaviours, though clear areas for program improvement are also evident when comparing changes across all measurements occasions [20].

Results for the primary outcome show that students largely retained their intentions to use quality first aid strategies. In our previous report on these measures at post-training, the tMHFA students showed statistically significant improvements on both the helpful and unhelpful intentions towards both John and Jeanie, though effects were larger for the John vignette (*d* = 0.58 helpful, *d* = 0.41 unhelpful), and smaller for Jeanie (*d* = 0.50, *d* = 0.15). In the current study, the significant improvements in the helpful and unhelpful intentions were still apparent for the John vignette, but not for the Jeanie vignette. A booster session with a particular focus on re-learning the tMHFA action plan and enacting it for peers with symptoms of anxiety, would likely improve students’ outcomes in this area.

Students who received the tMHFA program reported more confidence helping a peer with depression and suicide risk, or a peer with social anxiety/phobia, 12 months after their training than students who received the PFA program. This finding is consistent with other MHFA training evaluations which have found that confidence in providing assistance for MHPs significantly improves after the program and is retained over 12 months [39].

Similarly, tMHFA students rated a significantly greater number of adults as helpful for a peer like John or Jeanie, and more positive help-seeking intentions if they were to experience a problem like John or Jeanie’s, than PFA students. These changes were maintained at 12-month follow-up, suggesting that some important barriers to help-seeking may have shifted as a result of the program. By encouraging early help-seeking, tMHFA may contribute to better mental health outcomes for adolescents experiencing MHPs.

Outcomes on measures of stigmatising attitudes were weaker at follow-up compared to those found at post-training, especially for the John vignette. Our previous report [20] found that stigmatising attitudes were significantly reduced among tMHFA students across all subscales in response to the John vignette, though only on *weak-not-sick* and *would not tell anyone* for the Jeanie vignette. In the current study, only the *would not tell anyone* subscale showed a significant group x time interactions, across both vignettes. The literature on stigma-reduction interventions tends to rely on short-term follow-up, so understanding why effects drop off at 12-months is not well explored. A 2015 review of stigma-reduction interventions [40] found that of six controlled trials implemented in schools, the longest follow-up was 5-months. A 2018 review [41] found that combined contact-education interventions (stigma reduction strategies also used by tMHFA) tended to show small-to-medium effects post-training and non-significant outcomes at follow-up. The *would not tell anyone* item, which showed a statistically significant reduction at one year follow up in this study, is an especially important measure of willingness to disclose a mental health problem to others, and forms a predominant barrier for help-seeking [42].

Although this trial was adequately powered to detect effects on the primary outcome, the secondary outcomes, which related to the quality of behaviours provided or received rather than intentions towards the vignettes, were evaluated in a much smaller subset of participants who had reported the relevant first aid experiences. The *quality of first aid provided to a peer* measure, for example, required students to have reported: i) contact with a peer needing help, ii) providing help to that peer, and iii) completion of the quality of first aid measure to a standard acceptable for analyses, at both baseline and follow-up. The subsample of students reporting these three outcomes, was much smaller compared to our complete sample at baseline. As such, confidence intervals on measurement of first aid behaviours were wide. Had a larger sample reporting first aid behaviours been available, our small effect size of *d* = 0.17, may have been statistically significant, given that this effect was consistent with the size of the (significant) effects reported for the vignette measures of first aid intentions, which used the full sample.

The proportion of students reporting contact with a peer with a mental health problem reduced over time in both groups. This is an unexpected finding, but may be explained by either discontinuation in surveys overtime, or because of the changing nature of social contact from Year 10, where cohorts are kept in more structured subject groups, to Year 11, where their final school certificate subjects begin and create more disjointed timetables and cohorts.

The proportion of students offering help to a peer in the current study (81% at baseline, 88% at follow up across the whole sample) lies between two other studies reporting on MHFA training in Australian tertiary students which found 65% [43] and 92% [44] of trainees respectively reported applying their skills to someone in need, when surveyed up to 24 months after receiving training. As this is the first study to evaluate MHFA training in secondary students, only future research will be able to determine if the level of helping behaviours in the current sample is broadly representative of the rates of adolescent help-giving. Observational studies of adolescent help-giving are quite rare (Subasinghe, Morgan, Paxton, Hart, *under review*), and the field could benefit from empirical research that gauges naturalistic help-giving behaviours among adolescents. But perhaps even more important than improving the *quantity* of help given, is improving the *quality* of that help.

Our study is one of the first to be able to report on receipt of first aid among a sample who had received MHFA training, and the first to our knowledge, to provide findings for a sample of adolescents. In the current study 53 students reported having had a mental health problem or crisis at both baseline and follow-up, had received help from a peer at both timepoints, and reported survey data of sufficient quality to be included in analyses. The reduction in the available data for these measures was primarily a function of the reasonably high rate of discontinuation of students in this study, rather than a low rate of help-giving among peers. Nevertheless, compared to the complete dataset, there was a much smaller sample reporting on the quality of first aid behaviours over time, and thus it is likely these analyses were underpowered to detect small effects. On measures of both quality of help provided to a peer, and of the quality of help received, the group x time interactions were not statistically significant. Changes in scores, however, were in the expected direction with tMHFA students showing small gains on the measure of helpful first aid behaviours offered to a peer, over the PFA students, at follow-up.

One explanation for the null result on first aid behaviours provided to a peer is that the program was not effective in changing first aid behaviours. Whilst this remains a possibility, taken with the results on measures of quality of first aid intentions, confidence, and the *would not tell anyone* stigma subscale, along with our previously reported qualitative results from students and schools [45] which described a high level of engagement and satisfaction with the program, it seems less likely than the alternative explanation that the sample reporting first aid provision was underpowered to detect small but significant effects. Students receiving tMHFA reported the appropriate level of knowledge and beliefs to be able to enact helpful first aid behaviours one year after receiving the program, however, only future research with a much larger sample reporting first aid behaviours at follow-up, will provide more precision and clearer evidence of whether the training program is associated with positive changes in behaviour or not.

Our finding that both females and those with higher K6 scores were more likely to report helpful behaviours is consistent with previous literature that suggests those with existing MHPs and females are most likely to provide assistance [46]. The mechanism underlying this is, however, unclear. It is possible that those who have experienced psychological distress are more likely to recognise it in others, or to have higher levels of empathy for suffering in their peers and therefore more motivation to provide support. This reinforces the notion that whole-of-school community training is important in equipping cohorts with the necessary skills to support their peers, given that selective training— for example, of a small leadership student group—may miss those with MHPs who are most likely to provide assistance to their peers. However, it is concerning that the most vulnerable students are also those who are more likely to shoulder the burden of mental health support for their peers, and special care should be taken within school communities to ensure extra staffing and resources are provided by wellbeing/pastoral and counselling staff to not only support those seeking help for their own distress, but also those who are providing assistance to their peers. Having an organisation like Mental Health First Aid Australia provide policy and implementation guidance to schools, in concert with local mental health services who often help deliver the tMHFA curriculum as trained MHFA instructors, is especially helpful in this regard, as wrap-around services can support the whole school community with more mental health expertise than when mental health curriculum is delivered by regular classroom teachers in isolation.

### Limitations

This study had many strengths, including the robust CRXO design, well matched active comparator intervention, and use of validated measures. However, there were also important limitations that need to be considered alongside the findings. A primary limitation was the rate of discontinuation among students, from baseline through to 12-month follow-up.

Although secondary schools are widely considered a useful context for capturing follow-up measures, attrition in the current sample was relatively high (45.5% over the course of the study). Across the two groups, 56% of tMHFA students and 53% of PFA students returned the 12-month survey. Although the rate of providing help to a peer was high among students in this trial, the attrition rate reduced the size of our sample who reported first aid experiences, and when combined with the small effect sizes for behaviour change, this limited our ability to produce robust analyses on the measures of first aid behaviours provided and received. We hypothesise that discontinuation among students was due to a number of important factors including: school-based administration, inability of the research team to contact students directly, and the repetitive text-based nature of the measures with no incentivisation of students. As reported in our previous paper [20], discontinuation may have been exacerbated by our study design and ethics approval which required student anonymity and disallowed gathering of personal contact details directly from students which would have allowed the research team to remind individuals of the opportunity to participate. Instead, we relied on the participating schools to schedule survey sessions during regular class time, and if students were absent, for teachers to follow-up with these individuals. Some schools were able to complete these tasks very methodically, while other schools were difficult to communicate with and had very low response rates. For some of our schools, there seemed to be poorer engagement with the research processes once the perceived benefit had been received (i.e. the training was complete). Nevertheless, one comparable Australian study—an evaluation of the Youth Aware of Mental Health program in 18 schools with 556 Year 9 students [47]—reported an attrition rate of 64% at 6-months follow-up, which suggests that the attrition rate in the current study was not as high as might be expected. Further, despite discontinuation rates in the current study, our ITT analyses do provide robust models for the vignette measures using the larger sample of students.

Other important limitations of this research are that our findings are not widely generalisable to other populations due to our restricted sample of schools and that we were unable to measure dose or fidelity. We sampled four diverse schools in the state of Victoria, but all were above the mean on the index of socio-educational advantage and attended by predominantly White, English-speaking students. Further research with a larger and more diverse sample of schools would provide more generalisable results. In addition, gathering data on how many of the training sessions students attended, and whether each instructor adhered to the training protocols, would help ascertain whether dose and fidelity are associated with better outcomes.

### Future directions

Compared to post-training findings, retention of gains in knowledge and reductions in stigma dropped off over the year, suggesting that students could benefit from a short ‘booster’ session to help re-learn the key messages of the program. Re-learning through multi-session interventions has been shown to be effective and likely to result in long-term retention of skills and knowledge [48]. Having first aid training across the lifespan would also aid in this retention, but unlike the booster session, also offer the benefit of an updated curriculum relevant to novel contexts. Many universities now offer the Youth MHFA program to their undergraduate students, for example, to aid in the transition from secondary to tertiary education and the emergence of MHPs during this time [43, 44]. Further, there has also been the introduction of a teen MHFA program for students in Years 7 to 9 (those aged 12–15 years), which focuses on the skills and knowledge needed for younger adolescents who are making the transition from primary to secondary schooling and often see the adolescent-onset disorders emerge for the first time [4]. If students were to receive training at each important schooling transition (Year 7, Year 10 and when commencing tertiary or vocational training), it is likely that the requisite knowledge and attitudes for the provision of quality first aid to peers with MHPs or crises, would be retained over the very long term, giving the very best chance that supportive first aid behaviours are engaged and result in better mental health outcomes for young people.

## Conclusions

Findings on the efficacy of the teen Mental Health First Aid program in improving student knowledge, attitudes and behaviours were mixed. There was reliable evidence that tMHFA was associated with improvements in students’ quality of first aid intentions towards peers with depression and suicide risk, students’ confidence in helping peers with either depression or social anxiety/phobia, students’ willingness to disclose personal MHPs, and positive attitudes towards seeking help from adults. This study is the first to show effective stigma reduction in students one year after training. However, null results were found for improvements in the quality of first aid behaviours provided to a peer with a mental health problem, or those received from a peer. While it is possible that this was the result of student discontinuation reducing the sample size reporting first aid behaviours, only future research will be able to ascertain whether tMHFA is associated with reliable improvements in mental health first aid behaviours towards peers with MHPs. The program could benefit from a booster session and a focus on retaining the reductions seen in stigmatising attitudes at the post-measurement occasion.

## Supplementary Information

**Additional file 1.** Survey Vignettes.

## Data Availability

The datasets generated and/or analysed during the current study are not publicly available due limitations on consent to release the data but available from the corresponding author upon reasonable request.
