# Supplementary material for: teen Mental Health First Aid: 12-month outcomes from a cluster crossover randomized controlled trial evaluation of a universal program to help adolescents better support peers with a mental health problem
Source: BMC Public Health. 2022 Jun 10;22:1159. doi: 10.1186/s12889-022-13554-6 (PMC9185965; doi:10.1186/s12889-022-13554-6)
Supplement: Supplementary file 1 — Additional file 1. Survey Vignettes. [file 12889_2022_13554_MOESM1_ESM.docx]

## Supplementary Document 1

**Adapted Youth Mental Health Literacy Survey - Vignettes**

**John's Story**

John is a 16 year old who has been unusually sad and miserable for the last few weeks. He is tired all the time and has trouble sleeping at night. John doesn’t feel like eating and has lost weight. He can’t keep his mind on his studies and his marks have dropped. He puts off making any decisions and even day-to-day tasks seem too much for him. His parents and friends are very concerned about him. John says he will never be happy again and believes his family would be better off without him. John says he feels so desperate, he has been thinking of ways to end his life.

**Jeanie’s story**

Jeanie is a 16 year old living at home with her parents. Jeanie started at your school last year and you are the only friend she has made so far. She seems very shy and when you ask her why she doesn't make more of an effort, she says she would really like to make more friends but is scared that she’ll do or say something embarrassing when she’s around others. Although Jeanie’s schoolwork is OK she rarely says a word in class and becomes incredibly nervous, trembles, blushes and seems like she might vomit if she has to answer a question or speak in front of the class. At her house you have seen that Jeanie is quite talkative with her family, but becomes quiet if anyone she doesn’t know well comes over. She has stopped answering the phone and doesn't come to parties anymore. Jeanie says she knows her fears are unreasonable but she can’t seem to control them and this really upsets her.
